# Quantifying positional variation of retinal blood vessels in glaucoma

**DOI:** 10.1371/journal.pone.0193555

**Published:** 2018-03-15

**Authors:** Mengyu Wang, Qingying Jin, Hui Wang, Neda Baniasadi, Tobias Elze

**Affiliations:** 1 Schepens Eye Research Institute, Harvard Medical School, Boston, MA, United States of America; 2 Jilin University, Changchun, China; 3 Jilin University of Finance and Economics, Changchun, China; 4 Max Planck Institute for Mathematics in the Sciences, Leipzig, Germany; Soochow University Medical College, CHINA

## Abstract

We studied the relationship between major retinal blood vessel (BV) positions and glaucoma parameters based on pairs of Cirrus optical coherence tomography scans and Humphrey visual fields of 445 eyes from 445 glaucoma patients in our cross-sectional study. A trained observer marked the major superior and inferior temporal BV (artery and vein) positions on four concentric circles around the optic disc. Analysis of variance was performed to analyze the group differences of BV positions related to the factors of radius, BV type, myopia status and glaucoma stage. Subsequent t-tests were implemented to further study the effect of glaucoma stage on BV positions. The radial variations of BV positions were correlated to mean deviation and circumpapillary retinal nerve fiber layer thickness (cpRNFLT). We found significant main effects of BV type, radius and myopia status for superior and inferior BV positions and of glaucoma stage for superior BV positions (all p≤0.006) with significant superior artery nasalization in advanced compared to mild glaucoma on the two smallest circles (subsequent t-tests, p<0.05). In addition, MD (r = -0.10, p = 0.04) and cpRNFLT (r = -0.12, p = 0.02) were significantly correlated to the angle difference of superior arteries between the innermost and outermost circles. In conclusion, we demonstrated that peripapillary superior artery positions are significantly nasalized for advanced glaucoma.

## Introduction

Positional shifts of major retinal blood vessels (BVs) with glaucoma progression are relevant for two reasons. First, as major BVs are clearly visible on fundus images regardless of glaucoma severity, possible systematic shifts over glaucoma progression could be used in clinical diagnosis. Second, as major temporal BV positions correlate with the peaks of retinal nerve fiber layer thickness [1, 2], it has been proposed to use major temporal artery positions on a circle with radius of 1.73mm around the optic nerve head (ONH) to adjust circumpapillary retinal nerve fiber layer thickness (cpRNFLT) norms for optical coherence tomography measurements [3]. However, if major temporal artery positions would substantially shift with glaucoma progression, it would be important to quantify the BV shifts and to explicitly consider them in order to use BV positions in eyes with severe glaucoma as anatomical markers for pre-disease cpRNFLT peaks.

Prior research has shown the position of the central retinal vessel trunk (CRVT) within ONH (i.e., the exit position of the retinal BVs) to be associated with the patterns of glaucoma [4, 5]. In particular, Jonas and Fernández [6] have shown the position of the CRVT to be correlated with the pattern of glaucomatous rim loss. In addition, Huang et al. [7] have shown patterns of perimetric loss to be associated with CRVT position, that is eyes with severe glaucomatous visual field loss but a remaining central island of vision are likely to have the CRVT in the temporal optic disc region. Most recently, CRVT location has been shown to be exclusively correlated to central vision loss quantitatively [8]. Besides aforementioned cross sectional studies, Varma et al. [9] have shown that the retinal BVs within the ONH shift over time due to glaucoma progression in a longitudinal study. In a seminal recent work, Radcliffe et al. showed that retinal BVs shift not only inside but also outside the ONH with functional glaucoma progression [10].

In this work, we aim to quantify the variation of major retinal BV position outside ONH among glaucoma patients. More specifically, we investigate whether there are quantitative relationships between BV position and glaucoma diagnostic parameters.

In particular, we consider the assumption that glaucoma progression is frequently accompanied by more cpRNFLT thinning on the temporal side of the ONH compared to the nasal side, which in turn may “drag” the BVs inside the ONH to the nasal side [9, 10]. Therefore, we hypothesize a nasalization of BVs outside the ONH that gets weaker with increasing eccentricity. More specifically, we hypothesize that the BV positions outside the ONH are more nasalized in more severe glaucoma and the nasalization due to glaucoma severity gets weaker with respect to larger distance from the ONH.

## Materials and methods

This retrospective cross-sectional study was approved by the institutional review board (IRB) of Massachusetts Eye and Ear (MEE). The IRB waived the need for informed consent because of the retrospective nature of this study. This study adheres to the Declaration of Helsinki and all federal and state laws.

### Participants and data description

The OCT ONH scans and the accompanying visual fields (SITA Standard 24-2 protocol) of all patients who presented at MEE glaucoma service between 2011 and 2014 were initially selected and transferred from the machines (Humphrey Field Analyzer HFA-II and Cirrus HD-OCT, Software version 6.5, Carl Zeiss Meditec AG, JENA, Germany). The data selection criteria for visual fields were fixation loss ≤ 33%, false negative rates ≤ 20% and false positive rates ≤ 20%. The data selection criteria for Cirrus OCT scan (Optic Disc Cube protocol with A-scan resolution 200×200 within an area of 6 mm×6 mm) were signal strength ≥ 6 and within one year from the visual field measurement. If more than one measurement per eye met the criteria, the most recent measurement was selected. If both eyes of a patient met the selection criteria, only one eye was included randomly to avoid potential bias of data samples. As such, we had 2,161 pairs of OCT and visual field measurement of 2,161 eyes from 2,161 patients which passed the initial data reliability check.

### Data processing

The Cirrus OCT scan of ONH is centered around the optic disc with an area of 6 mm×6 mm. The ONH center was determined by the Cirrus machine as the centroid of Bruch’s membrane opening [11]. The retinal thickness color maps and the corresponding fundus images of each eye were centered based on the ONH center. OCT scans with ONH centers which deviated more than 0.3 mm in horizontal or vertical direction from the fundus image center were excluded. To ensure the availability of data over the complete area for each centered image, the edges of the centered images were removed by 0.3 mm that resulted in an image area of 5.4 mm×5.4 mm. To ensure the reliability of cpRNFLT measurement, we excluded eyes with OCT scans that had missing data of cpRNFLT measurements, which were denoted by black areas on the cpRNFLT color plot. Moreover, OCT scans with motion artifact (BV shifts of more than one BV diameter or a visible shift within ONH which were visually checked by a trained observer) were also excluded. In addition, eyes with incomplete diagnostic details in the patient’s medical record and with cataract (nuclear sclerosis 3+ or worse) were excluded.

### Vessel tracking

**Fig 1:**
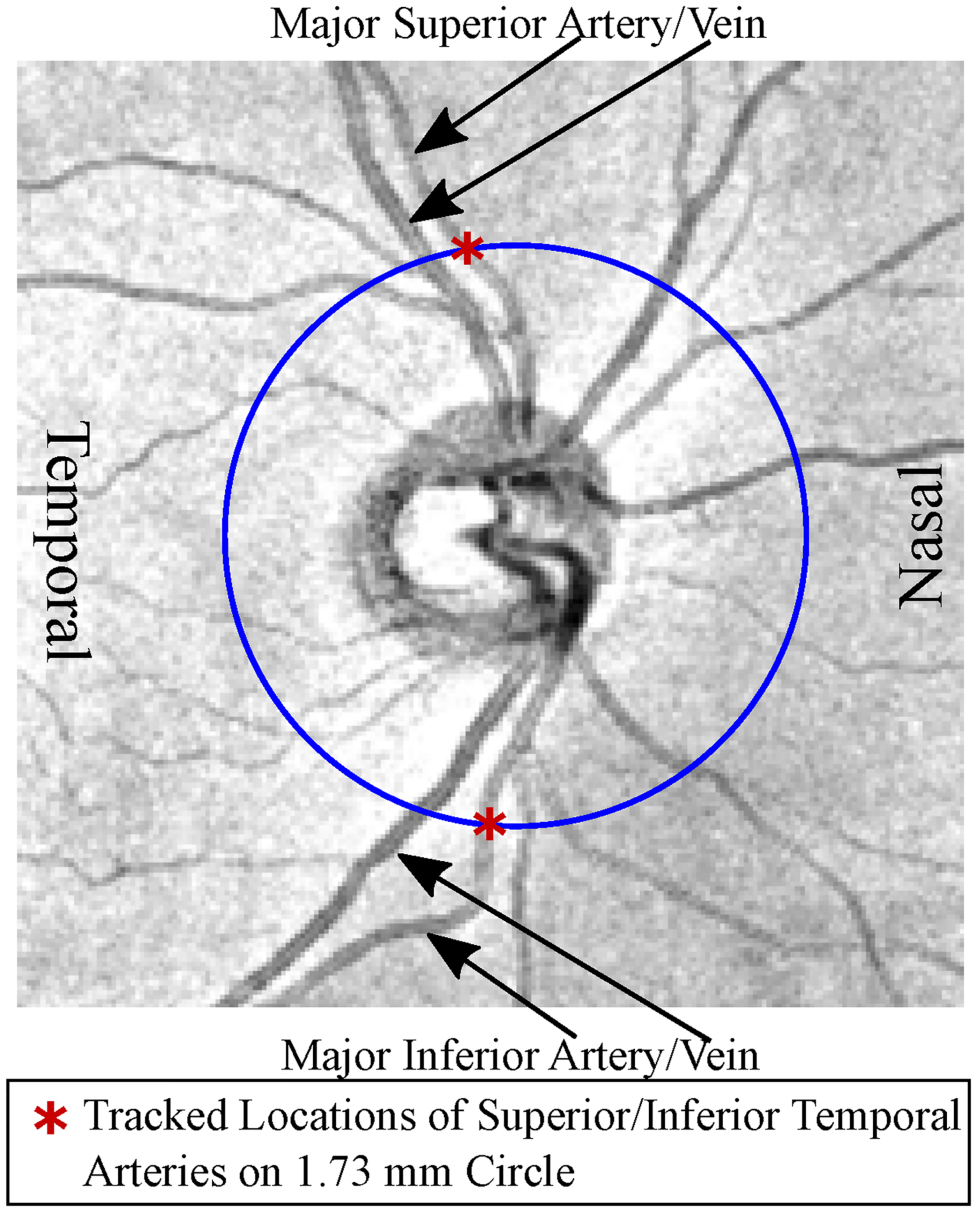
Example of fundus image of a right eye with tracked major superior temporal artery and inferior temporal artery positions on the circle with 1.73 mm radius.

For BV tracking, we developed a custom software in the programming language R (version 3.2.2, R Foundation, Vienna, Austria) [12]. A trained observer marked the positions of the major superior and inferior temporal arteries and veins on four concentric circles around the ONH center with radii 1.23, 1.73, 2.23, and 2.70 mm on fundus image, respectively, on the OCT fundus image. Fig 1 shows an example of the major superior and inferior temporal artery positions on the circle with 1.73 mm radius, which is the standard circle of the Cirrus cpRNFLT printout that is frequently used by clinicians for diagnostic purposes. We used the coordinate system of Cirrus device which defines the angular position of zero as horizontal line towards temporal direction and calculate angles clockwise/counterclockwise for right/left eyes.

### Statistical analysis

All statistical analyses were performed by R platform [12]. Analysis of variance (ANOVA) was performed to test whether there are significant group differences of major BV angular positions with respect to the following factors: radius (from innermost to outermost circles), BV type (artery or vein), myopia status (myopia: spherical equivalent < 0 diopter, non-myopia: spherical equivalent ≥ 0 diopter) and glaucoma stage (mild [visual field mean deviation (MD) ≥ -6 dB] or advanced [MD < -6 dB] glaucoma). The spherical equivalent was calculated by adding half cylinder power to the sphere power, which was measured by subjective refraction. The ANOVA was implemented to compare the superior and inferior major BV positions between mild and advanced glaucoma, respectively. To further elucidate the relationships between major BV position and glaucoma stage, we performed subsequent data analyses of the significant main effects to check our specific hypotheses. In particular, the mean angular positions of superior and inferior BVs on the circles with each radius between mild glaucoma and advanced glaucoma were compared by t-test [13]. In addition, we evaluated the correlation between BV positions and MD as well as cpRNFLT. Furthermore, we performed linear regression to examine the association between the superior artery positions that are significantly different between mild and advanced glaucoma and mean total deviation of the inferior hemifield of visual field. Lastly, we also applied linear regression to study the association between the angle difference between the innermost and outermost position of retinal BVs and MD and cpRNFLT, respectively.

## Results

As shown in Table 1, 1,480 of the initial selected 2,161 eyes were excluded due to OCT scanning artifacts (see Methods). In addition, 122 and 124 eyes were excluded due to incomplete diagnostic data in the patient’s medical record and cataract as a possibly confounding disease, respectively. Ultimately, 445 eyes passed our selection criteria and are used in our data analyses.

**Table 1:** Results of the iteratively applied data selection criteria (see Methods). Note that the number of excluded eyes is always relative to the number of remaining eyes in the respective previous table row.

Criterion	Eyes Excluded	Eyes Remaining
Initially Transferred from Machine		2161
Out of Center	221	1940
Motion Artifacts	1082	858
OCT with Missing Data	167	691
Incomplete Diagnostic Data	122	569
Cataract	124	445

Table 2 shows the demographics and diagnostic details for the 445 patients that passed our data reliability criteria for mild and advanced glaucoma. In addition, Table 2 also shows the blood vessel positions of superior and inferior arteries and veins for mild and advanced glaucoma.

**Table 2:** Descriptive statistics of the demographics, detailed diagnostics and blood vessel positions including SAA, SVA, IAA and IVA on the four concentric circles with radii of R_1_: 1.23 mm, R_2_: 1.73 mm, R_3_: 2.23 mm and R_4_: 2.70 mm for mild (MD ≥ -6 dB) and advanced (MD < -6 dB) glaucoma. MD: mean deviation in decibel (dB); cpRNFLT: circumpapillary retinal nerve fiber layer thickness; OAG: primary or secondary open angle glaucoma; ACG: primary or secondary angle closure glaucoma; MMG: mixed mechanism glaucoma; SAA: superior artery angle; SVA: superior vein angle; IAA: inferior artery angle; IVA: inferior vein angle.

Glaucoma Severity	Mild	Advanced
Age (years)	58.6±13.1	61.3±12.5
Sex (M/F)	175/206	27/37
MD (dB)	−1.6±1.8	−11.5±5.2
cpRNFLT (*μ*m)	83.7±11.9	65.7±13.1
OAG	153	47
ACG	2	3
MMG	6	3
Suspect	220	11
SAA at R_1_ (°)	78.94±12.53	82.42±13.94
SAA at R_2_ (°)	72.8 ±12.13	75.75±12.77
SAA at R_3_ (°)	67.88±11.12	70.09±11.98
SAA at R_4_ (°)	63.74±10.39	65.83±11.56
SVA at R_1_ (°)	82.28±15.23	83.28±14.95
SVA at R_2_ (°)	75.38±14.48	76.01±13.99
SVA at R_3_ (°)	69.81±13.24	70.48±13.42
SVA at R_4_ (°)	65.59±12.6	66.04±12.66
IAA at R_1_ (°)	278.17±15.41	275.54±16.01
IAA at R_2_ (°)	284.94±13.97	282.55±14.82
IAA at R_3_ (°)	290.36±12.42	288.13±11.99
IAA at R_4_ (°)	294.43±11.59	292.92±10.51
IVA at R_1_ (°)	272.52±16.93	274.06±15.62
IVA at R_2_ (°)	279.18±15.5	279.97±14.98
IVA at R_3_ (°)	284.15±14.72	285.23±14.11
IVA at R_4_ (°)	287.91±14.16	289.42±13.79

As shown in Table 3, there were significant main effects of BV type (p<0.001), radius (p<0.001), myopia status (p<0.001) and glaucoma stage (p = 0.006) on the major BV positions in the superior sector and significant main effects of BV type (p<0.001), radius (p<0.001) and myopia status (p<0.001) but no significant effect of glaucoma stage (p = 0.48) on the major BV positions in the inferior sector.

**Table 3:** ANOVA results of the impact of blood vessel type, radius, myopia status (myopia: Spherical equivalent < 0 diopter, non-myopia: Spherical equivalent ≥ 0 diopter) and glaucoma stage (mild: MD≥-6 dB, advanced: MD< -6 dB) on the major blood vessel positions in the superior and inferior sectors, respectively. ANOVA: analysis of variance; MD: mean deviation. Significant results (P<0.05) are marked by asterisks.

Hemifield	Superior (P Value)	Inferior (P Value)
Vessel Type	p< 0.001*	p< 0.001*
Radius	p< 0.001*	p< 0.001*
Myopia Status	p< 0.001*	p< 0.001*
Glaucoma Stage	p = 0.006*	p = 0.48

**Fig 2:**
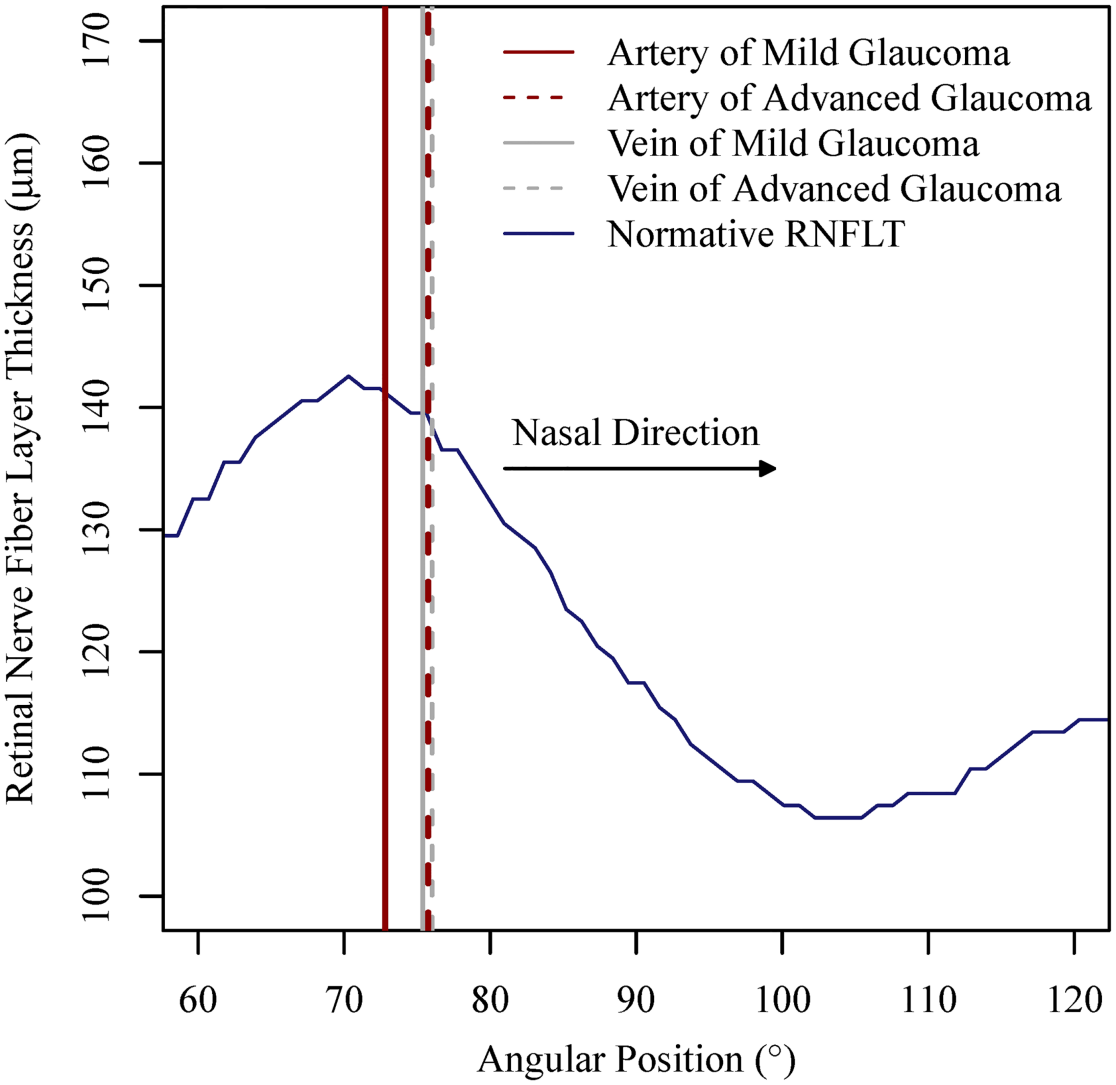
Comparison of mean angular positions of superior BVs between mild and advanced glaucoma on the circle with 1.73 mm radius (i.e., Cirrus standard measurement circle). As a spatial reference, the Cirrus cpRNFLT norm was added (blue line). Here, the norm for the age of 52 years is shown. The absolute cpRNFLT values decrease with age, but the peak location remains stable.

Fig 2 illustrates the mean angular positions of superior arteries and veins in mild glaucoma compared to advanced glaucoma on the circle with 1.73 mm radius. As a spatial reference, we added the population based circumpapillary retinal nerve fiber layer thickness (cpRNFLT) norms from the Cirrus machine (blue line). In the figure, the norm for the age of 52 years is shown. For different ages, cpRNFLT is scaled up (younger ages) or down (higher ages), but the peak locations used here as spatial references, remain at the same positions. Superior BVs in advanced glaucoma were more nasal and more distant from the normative cpRNFLT peak than in mild glaucoma. The nasalization of arteries from mild glaucoma to advanced glaucoma was more notable than veins.

**Fig 3:**
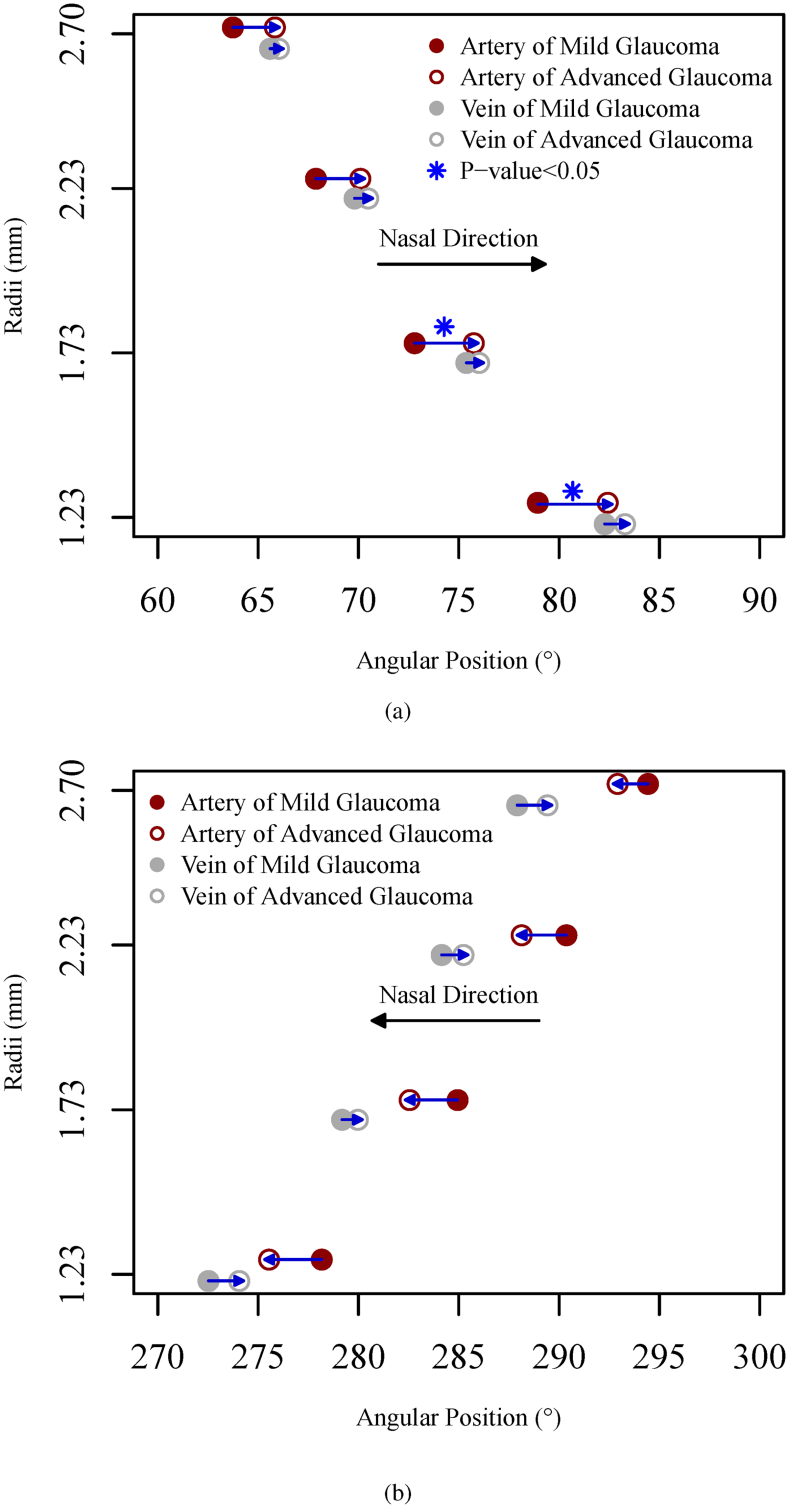
Comparison of mean angular position of superior (a) and inferior (b) BVs between mild and advanced glaucoma on the four concentric circles. Asterisks denote a significant positional difference between mild and advanced glaucoma.

Given the significant main effects of BV type and radius, subsequent data analyses to compare the BV positional differences between mild and advanced glaucoma stratified by BV type and radius were performed. Fig 3(a) and 3(b) show the positional differences of superior and inferior BVs between mild and advanced glaucoma on the four concentric circles, respectively. The superior positions of BV (artery and vein) and inferior positions of artery in advanced glaucoma tended to be more nasal than in mild glaucoma on all 4 circles, while only the positional differences of superior arteries between mild and advanced glaucoma on the circles with radii of 1.23 mm (p = 0.03) and 1.73 mm (p = 0.04) were statistically significant. More specifically, superior arteries in advanced glaucoma were located in more nasal positions than in mild glaucoma for 3.49°, 2.95°, 2.21° and 2.10° from the innermost to outermost circles, and inferior arteries in advanced glaucoma were located in more nasal positions than in mild glaucoma for 2.63°, 2.39°, 2.23° and 1.51° from the innermost to outermost circles. In contrast to arteries, the positional differences of veins between mild and advanced glaucoma were less distinct. Specifically, superior veins in advanced glaucoma were located in more nasal positions than in mild glaucoma for 1.01°, 0.63°, 0.68°, and 0.44° from the innermost to outermost circles, and inferior veins in advanced glaucoma were located in more temporal positions than in mild glaucoma for 1.53°, 0.79°, 1.09° and 1.51° from the innermost to outermost circles.

**Fig 4:**
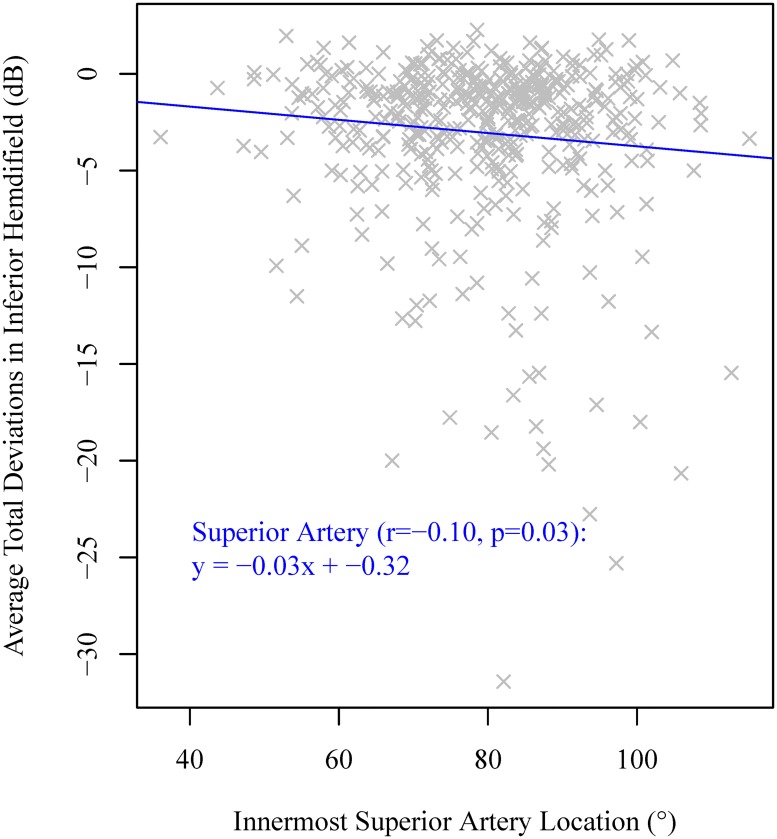
Linear regression from the innermost superior artery positions to the average total deviation in inferior hemifield of the visual field.

As shown in Table 4, there were no significant correlations between BV positions on any of the four concentric circles and MD, as well as cpRNFLT. The superior artery position on the circle with 1.23 mm radius was significantly correlated (r = -0.10, p = 0.03) to the average total deviation of the inferior hemifield of the visual field (see Fig 4). In addition, the superior artery position on the Cirrus standard circle with 1.73 mm radius was marginally significantly correlated (r = -0.09, p = 0.05) to the average total deviation of the inferior hemifield of the visual field.

**Table 4:** The correlation results between the SAA, SVA, IAA and IVA on the four concentric circles with radii of R_1_: 1.23 mm, R_2_: 1.73 mm, R_3_: 2.23 mm and R_4_: 2.70 mm and glaucoma diagnostic parameters including MD and cpRNFLT. SAA: superior artery angle; SVA: superior vein angle; IAA: inferior artery angle; IVA: inferior vein angle; MD: mean deviation; cpRNFLT: circumpapillary retinal nerve fiber layer thickness. Significant results (P<0.05) are marked by asterisks.

	MD		cpRNFLT	
	Correlation	P Value	Correlation	P Value
	Coefficient		Coefficient	
SAA at R_1_	-0.08	0.09	-0.08	0.09
SAA at R_2_	-0.07	0.14	-0.05	0.25
SAA at R_3_	-0.03	0.50	-0.01	0.77
SAA at R_4_	-0.03	0.59	-0.01	0.80
SVA at R_1_	-0.03	0.54	0.02	0.70
SVA at R_2_	-0.02	0.62	0.03	0.55
SVA at R_3_	-0.02	0.71	0.04	0.41
SVA at R_4_	-0.01	0.89	0.04	0.36
IAA at R_1_	0.07	0.15	-0.05	0.33
IAA at R_2_	0.07	0.15	-0.07	0.12
IAA at R_3_	0.05	0.25	-0.09	0.06
IAA at R_4_	0.05	0.27	-0.08	0.11
IVA at R_1_	-0.03	0.56	0.01	0.87
IVA at R_2_	-0.01	0.79	0.01	0.82
IVA at R_3_	-0.02	0.72	-0.01	0.84
IVA at R_4_	-0.03	0.55	-0.02	0.67

**Fig 5:**
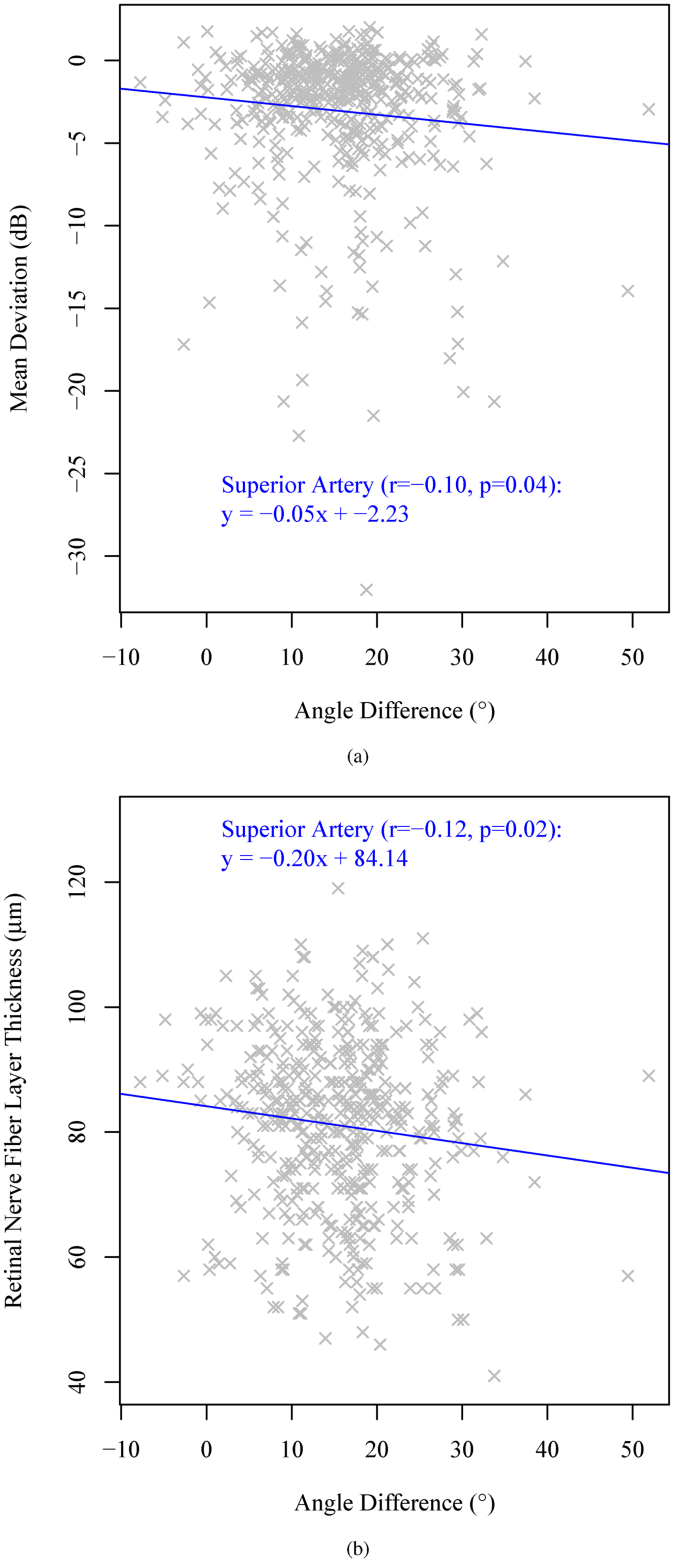
Linear regressions from the angle difference between the innermost and outermost positions of superior artery to (a) the mean deviation and (b) the cpRNFLT.

Fig 5(a) and 5(b) show the linear regressions from the angle difference between the innermost and outermost positions of superior arteries to the mean deviation and the cpRNFLT on the standard diagnostic circle on Cirrus with 1.73 mm radius, respectively. The angle difference of between the innermost and outermost positions of superior arteries was negatively correlated to MD significantly (r = -0.10, p = 0.04) and positively correlated to cpRNFLT significantly (r = -0.12, p = 0.02). There were no significant correlations between angle difference of other BVs and MD, as well as cpRNFLT as detailed in Table 5.

**Table 5:** The correlation results between the differences between the innermost and outermost locations of blood vessels including SAA, SVA, IAA and IVA and glaucoma diagnostic parameters including MD and cpRNFLT. SAA: superior artery angle; SVA: superior vein angle; IAA: inferior artery angle; IVA: inferior vein angle; MD: mean deviation; cpRNFLT: circumpapillary retinal nerve fiber layer thickness. Significant results (P<0.05) are marked by asterisks.

	MD		cpRNFLT	
	Correlation	P Value	Correlation	P Value
SAA Difference	-0.10	0.04*	-0.12	0.02*
SVA Difference	-0.04	0.35	-0.03	0.47
IAA Difference	0.05	0.27	0.02	0.70
IVA Difference	-0.01	0.87	0.05	0.29

## Discussion

For the superior sector, there were significant main effects of BV type, radius, myopia status and glaucoma stage on the major BV positions. For inferior sector, there were significant main effects of BV type, radius and myopia status but no significant main effect of glaucoma stage on the major BV positions. The superior BVs (arteries and veins) and inferior arteries in advanced glaucoma tended to be located in more nasal positions than in mild glaucoma, while the nasalization effect was only significant for superior arteries outside ONH for radii up to the Cirrus standard circle with 1.73 mm radius. In particular, the nasalization effect of arteries was more notable than that of veins while the nasalization effect of superior BVs was more notable than that of inferior BVs. Although peripapillary superior artery positions in advanced glaucoma were more nasal than in mild glaucoma, there were no significant correlations between the peripapillary superior artery positions and MD, as well as cpRNFLT. However, this does not rule out the possibility that there are individual shifts with glaucoma progression that would be observable in longitudinal measurements. The inter-individual BV positional variation might be too large to detect these effects in our cross-sectional study. Furthermore, we demonstrated that the peripapillary superior artery positions were significantly correlated to average total deviation in inferior hemifield of the visual field.

The results plotted in Fig 3 suggest that the arteries and veins move closer for advanced glaucoma compared to mild glaucoma on population average level, which is in fact not the case on individual level. The standard deviations of angle difference between the innermost positions of superior arteries and vein for mild and advanced glaucoma are 14.67° and 16.19°, which did not support the hypothesis that the positions of arteries and veins move closer to each other due to glaucoma progression. However, we found that 40.1% and 50.0% of the patients in mild and advanced glaucoma had the innermost positions of superior arteries that were more nasal than superior veins. This may indicate a positional switch between superior arteries and veins during glaucoma progression.

Our findings are consistent with the prior research accomplishments which demonstrated the nasalization shift effect of BVs [9, 10]. In our study, although there was a significant main effect of glaucoma stage on BV positions, only peripapillary superior artery positions (up to the Cirrus standard diagnostic circle) are significantly more nasal in advanced glaucoma than in mild glaucoma. A common explanation of BVs shifts is that a focal loss of axons may induce a tractional force that leads to the BV shifts [10].

We observed that the outermost BV positions were barely affected by glaucoma severity in contrast to innermost BV positions. The angle difference of superior arteries was significantly correlated to MD and cpRNFLT, respectively. Therefore, the angle difference between the innermost and outermost positions of superior arteries can be used as a measure to alarm potential superior artery shift for each individual patient even in the absence of longitudinal data of aligned fundus images. By recognizing possible BV nasalization due to glaucoma severity, we can potentially avoid the deceptive phenomenon of areas of improvement in glaucoma monitoring [14] due to the cpRNFLT peak position changes related to BV shifts in clinical practice.

There are limitations in our study. First, the correlations between superior artery nasalization and glaucoma diagnostic parameters were quite weak due to the confounding factors of large variance of individual BV anatomy. Future studies are needed to compare the inter-eye BV positions and their relationships with glaucoma diagnostic parameters to minimize the effect of individual BV anatomy to render better correlations. Second, we do not have longitudinal data in this study to evaluate the relationship between BV nasalization and functional progression rate. We might find that the BV nasalization effects might not be limited to peripapillary superior artery positions. Lastly, the nasalization of BVs in superior/inferior hemifield might be related to specific visual field sectors [15] or representative patterns [16] instead of the entire hemifield. Future studies are needed to investigate possible relationship between BV nasalization and visual field loss patterns.

To sum up, the peripapillary superior artery positions are significantly nasalized for advanced glaucoma compared to mild glaucoma, while the blood vessel locations that are further from the ONH barely change in advanced glaucoma compared to mild glaucoma. However, there are no significant correlations between any of the BV locations and glaucoma diagnostic parameters including MD and cpRNFLT, which might suggest that the nasalization effect of glaucoma severity is non-linear rather than linear. Furthermore, we found that the angle difference between the innermost and outermost locations of superior artery is significantly correlated to MD and cpRNFLT, respectively.
